# Acid Extract of the Duodenal Mucous Membrane as a Remedy in Diabetes Mellitus

**Published:** 1906-02-10

**Authors:** 


### ACID EXTRACT OF THE DUODENAL MUCOUS MEMBRANE AS A REMEDY IN DIABETES MELLITUS.

Dr. Jno. Hill Abram [1] reports a therapeutic experiment of much interest—namely, the use of an acid extract of the duodenal mucous membrane in the treatment of diabetes mellitus. The procedure is based on the work of Bayliss and Starling, who have shown that the contact of hydrochloric acid with the duodenal epithelium causes in the latter the production of a substance which stimulates the pancreas to secretion. With this increased activity of the pancreas there is an added flow of lymph in the vessels passing from the gland, and thus it may be anticipated—though it is not proved—that there is an increase in the internal secretion of the pancreas. There can be no doubt that the pancreas plays an important part in the assimilation of carbohydrates, and hence an agent which stimulates its activity may reasonably be expected to exert some influence in cases of diabetes. By some such line of reasoning the conclusion is reached that an acid extract of the duodenal mucous membrane has a *prima facie* case in its favour. At the same time it is recognised that in different cases there may well be disturbances in different parts of the carbohydrate-assimilation mechanism, and thus it is not improbable that a remedy which succeeds in some cases may altogether fail in others. In this cautious spirit Dr. Abram records his experience with the acid extract of duodenal mucous membrane. The first case was that of a man of 25 years, with typical symptoms of diabetes mellitus; the urine contained both acetone and di-acetic acid. The acid extract was given from January to May 1905, when the urine had become quite free from sugar, and the polyuria had ceased. The second patient was a boy of seven years, passing a daily average of 2,685 grains of sugar. Under the influence of appropriate diet and of antipyrin and duodenal extract the sugar entirely disappeared in the course of some eight weeks. In a third patient a similar experience was noted. Obviously these cases are too few in number to form a basis for any generalisation, or indeed for any very confident statement as to the influence of the extract in the instances quoted. There are opportunities for accident and coincidence which must not be forgotten. Further, in two other cases recorded by Dr. Abram the extract produced no appreciable effect, though in each it must be admitted that it enjoyed but a brief trial. With all the necessary qualifications and allowances, however, the positive facts advanced by Dr. Abram are sufficient to justify further trials on the line he has adopted. Experience of the action of drugs in cases of diabetes, more especially in the more rapid form of the disease met with in young patients, is not particularly encouraging, and anything which promises to have some measure of therapeutic value ought to receive a fair and reasonable trial.

[1] The Lancet, Jan. 27, 1906.

